# Supplementary material for: Risk of Tuberculosis Caused by Fluticasone Propionate versus Budesonide in Chronic Obstructive Pulmonary Disease: A Nationwide Population-Based Study
Source: J Pers Med. 2022 Jul 21;12(7):1189. doi: 10.3390/jpm12071189 (PMC9321025; doi:10.3390/jpm12071189)
Supplement: Supplementary file 1 [file jpm-12-01189-s001.zip › jpm-1800919-supplementary.pdf]

Table S1. Baseline characteristics of unmatched study subjects

	Fluticasone propionate (n=38,628)		Budesonide (n=16,514)		p value
	n	%	n	%	
**Age** (years)					
Mean (SD)	64.80 (11.65)		62.47 (11.54)		<0.0001
40-49	4,584	11.87	2,590	15.68	<0.0001
50-59	8,203	21.24	4,174	25.28	
60-69	11,262	29.16	4,854	29.39	
70-79	10,571	27.37	3,716	22.50	
≥ 80	4,008	10.38	1,180	7.15	
**Sex**					0.0217
Male	21,494	55.64	9,364	56.70	
Female	17,134	44.36	7,150	43.30	
**Comorbidity**					
Bronchiectasis	3,482	4.59	1,452	4.92	0.4036
Diabetes	13,648	17.98	5,215	17.66	<0.0001
Hypertension	24,277	31.97	9,445	31.99	<0.0001
Heart failure	9,344	12.31	3,260	11.04	<0.0001
Stroke	9,530	12.55	3,569	12.09	<0.0001
Chronic kidney disease	2,259	2.98	861	2.92	0.0031
Chronic liver disease	13,386	17.63	5,720	19.38	<0.0001
**CCI**					
Mean (SD)	3.38 (2.19)		3.01 (2.08)		<0.0001
<2	8,135	21.06	4,586	27.77	<0.0001
≥ 2	30,493	78.94	11,928	72.23	
**Bronchodilator**					
SABA	712	1.84	1,035	6.27	<0.0001
LAMA	5,793	15.00	2,231	13.51	<0.0001
LABA	29,621	76.68	12,134	73.48	<0.0001
LAMA/LABA	2,502	6.48	1,114	6.75	0.2431
**OCS prescription**					
Yes	32,400	87.25	13,602	84.65	<0.0001
No	4,733	12.75	2,467	15.35	
**OCS prescription day**					
Mean (SD)	13.84 (21.20)		10.98 (17.56)		<0.0001
**Interval from COPD diagnosis to index date**					
Mean (SD)	457.6 (829.7)		467.7 (905.8)		0.2010

CCI, Charlson Comorbidity Index; SABA, short-acting β-agonist; LAMA, long-acting muscarinic antagonist; LABA, long-acting β_2 agonist; OCS, oral corticosteroid.

Table S2. Hazard ratio of tuberculosis according to the ICS use in unmatched study subjects

Case (n, %)	ICS type		Univariate analysis		Multivariate analysis	
	Fluticasone propionate	Budesonide	HR (95% CI)	p value	HR (95% CI)	p value
Total	38,628	16,514				
Tuberculosis (-)	37,949 (98.24)	16,330 (98.89)	1.00		1.00	
Tuberculosis (+)	679 (1.76)	184 (1.11)	1.44 (1.22–1.70)	<0.0001	1.21 (1.02–1.44)	0.0027

Table S3. Hazard ratio of tuberculosis according to the ICS use

Variables	Univariate analysis		Multivariate analysis	
	Hazard ratio (95%CI)	*P* value	Hazard ratio (95%CI)	*P* value
Total	1.26 (1.04–1.53)	0.0180	1.28 (1.05–1.60)	0.0167
ICS cumulative dose (μg)				
0–15,000 (Q1)	0.97 (0.69–1.37)	0.8488	1.01 (0.69–1.48)	0.9523
15,001–60,000 (Q2)	1.16 (0.74–1.80)	0.5172	1.16 (0.74–1.81)	0.5168
60,001–225,000 (Q3)	1.28 (0.82–2.01)	0.2756	1.25 (0.79–1.97)	0.3344
> 225,000 (Q4)	1.83 (1.27–2.62)	0.0012	1.82 (1.27–2.62)	0.0012
